# Prevalence and Comorbidity of Atopic Dermatitis in Children: A Large-Scale Population Study Based on Real-World Data

**DOI:** 10.3390/jcm9061632

**Published:** 2020-05-28

**Authors:** Yolanda Gilaberte, Juan Blas Pérez-Gilaberte, Beatriz Poblador-Plou, Kevin Bliek-Bueno, Antonio Gimeno-Miguel, Alexandra Prados-Torres

**Affiliations:** 1 Department of Dermatology, Miguel Servet University Hospital, IIS Aragón, 50009 Zaragoza, Spain; ygilaberte@gmail.com; 2 Department of Dermatology, University of Zaragoza, 50009 Zaragoza, Spain; juanblasperezgilaberte@gmail.com; 3 EpiChron Research Group, Aragon Health Sciences Institute (IACS), IIS Aragón, Health Services Research on Chronic Patients Network (REDISSEC), Miguel Servet University Hospital, 50009 Zaragoza, Spain; bpoblador.iacs@aragon.es (B.P.-P.); sprados.iacs@aragon.es (A.P.-T.); 4 Teaching Unit of Preventive Medicine and Public Health, Miguel Servet University Hospital, 50009 Zaragoza, Spain; kbliek@salud.aragon.es

**Keywords:** atopic dermatitis, children, comorbidity, multimorbidity, prevalence, Spain

## Abstract

This study aimed at exploring atopic dermatitis (AD) prevalence in children and exhaustively analyzing their comorbidity. We conducted a descriptive analysis of their socio-demographic and comorbidity characteristics in the EpiChron Cohort (Aragón, Spain). Adjusted odds ratios (OR) were calculated for each comorbidity using logistic regression models. In total, 33,591 children had a diagnosis of AD, resulting in an overall prevalence of 15.5%. AD prevalence was higher in girls compared to boys, in 3–9-year-olds compared to children of other ages, and in Spanish children compared to those of other nationalities. Multimorbidity was present in 43% of children, with the most frequent chronic comorbidities being asthma (13.1%), psychosocial disorders (7.9%), and visual impairment (7.8%). Many diseases were, regardless of their prevalence, statistically associated with AD. The strongest associations (odds ratio (OR) (95% confidence interval (CI))) were found in asthma (2.10 (2.02–2.17)), allergic rhinitis (2.00 (1.91–2.10)), and irritable bowel syndrome (1.90 (1.56–2.31)). A better understanding of the array of comorbidities associated with AD in children might help improve their clinical management. Future longitudinal studies are encouraged to shed light on the potential underlying pathophysiological mechanisms involved in the identified associations.

## 1. Introduction

Atopic dermatitis (AD) is a chronic inflammatory skin disease of multifactorial etiology characterized by age-specific skin lesions, xerosis, and highly pruritic outbreaks. Usually starting in early childhood, AD significantly reduces patients’ quality of life [1,2] and increases healthcare resource use [3]. According to the estimates of the International Study of Asthma and Allergies in Childhood (ISAAC) [4], AD globally affects 15%–20% of children and 1%–3% of adults [5]. These prevalence figures are growing worldwide and show clear geographical variances; AD is more frequent in industrialized and high-income countries than in agriculture-based ones with low incomes [6,7]. Only one study to date exhaustively analyzed AD prevalence during childhood in Spain; it was conducted in 2008 using a population-based survey and concluded that AD affects around 30% of 6–7-year-olds [8].

Patients with AD are more susceptible to the development of allergic diseases and frequently suffer from multimorbidity (i.e., coexistence of more than one chronic condition). Multiple studies link AD during infancy to the future appearance of asthma and allergic rhinitis, with 80% of patients eventually developing either of them or both [9]. Food allergies occur in 35% of children with AD [10], and cutaneous infections and keratoconjunctivitis are more frequent than in the general population [11,12,13]. Psychiatric and psychological disorders, especially attention deficit hyperactivity disorder (ADHD), depression, or anxiety, are also more frequent in AD patients, possibly due to pruritus-induced lack of sleep, psychological stress, and high levels of proinflammatory cytokines [9]. Other comorbidities of varying nature such as cardiovascular diseases, type 2 diabetes, obesity, infections that go beyond cutaneous over-infection, malignancies (e.g., lymphomas), or autoimmune disorders (e.g., alopecia areata, lupus erythematosus, and inflammatory bowel disease) are also somehow linked to AD [12,14,15,16]. To the best of our knowledge, there are no large-scale population-based publications specifically analyzing the global comorbidity of AD during childhood.

Improving our knowledge on the comorbidity profile of children with AD is necessary for their optimal clinical management, which should also consider the concurrence of different comorbidities, and which could shed some light on the underlying pathophysiological mechanisms connecting AD to other diseases. The characterization of the comorbidity profile of this population group should focus not only on the identification of the most common diseases accompanying AD, but also on those that are systematically associated to AD regardless of their prevalence. The results obtained in this regard could help identify which comorbidities deserve special attention for the development of holistic, person-centered clinical strategies for AD patients.

The objective of this large-scale, population-based study is to determine the prevalence of AD in children under 18 years of age in a Mediterranean region of Spain, to exhaustively describe their most prevalent comorbidities, and to analyze which diseases display an increased risk of appearance when AD is present.

## 2. Experimental Section

### 2.1. Study Design and Population

We conducted a retrospective observational study in the EpiChron Cohort, which links socio-demographic and clinical data from all the users of the public health system of the Spanish region of Aragón [17]. This cohort is based on the information registered in the electronic health records (EHRs) and clinical–administrative databases of approximately 98% of the inhabitants of Aragón (reference population: 1.3 million people). Diagnoses are coded using the International Classification of Primary Care, First Edition (ICPC–1) for primary care health records, or the International Classification of Diseases, Ninth Revision, Clinical Modification (ICD–9–CM) for hospital health records. Diagnoses are subsequently grouped in expanded diagnostic clusters (EDCs) through the Johns Hopkins Adjusted Clinical Groups (ACG^®^) System [18] (version 11.0, The Johns Hopkins University, Baltimore, MD, US). A description of the cohort profile that served as the basis of more than 20 clinical–epidemiological studies on chronic diseases, as well as of the information sources and data curation procedures used, was published elsewhere [17].

For this study, we selected the individuals of the cohort aged 17 and under on 31 December 2015 (*n* = 216,291). Patients with AD were then identified through the appropriate diagnostic codes recorded in their primary (i.e., ICPC–1 code “S87”) and/or hospital (i.e., ICD–9–CM code “691´, excepting “691.0”) EHRs between 1 January 2015 and 31 December 2015. In Spain, AD is diagnosed clinically by dermatologists or pediatricians based on medical history, the morphology and distribution of skin lesions, and associated clinical signs [19]. Only patients living in Aragón and registered as users of the public health system during the year prior to the start of the study and during the complete one-year follow-up period were included.

The Clinical Research Ethics Committee of Aragón (CEICA) approved this study (Research protocol PI18/082) and waived the requirement to obtain the informed consent from patients given the epidemiological nature of the project, which used anonymized data.

### 2.2. Study Variables

For each subject, we analyzed the following variables: sex, age as of 31 December 2015, nationality, type of residence according to the type of administrative health area (urban, if 80% or more of the population of the area is concentrated in one of the municipalities, or rural for the rest), deprivation index of the area according to 26 socio-economic indicators ordered from least (Q1) to most (Q4) deprived areas [20], and all chronic disease diagnoses, as well as those of specific, acute conditions considered clinically relevant in AD (i.e., allergic rhinitis, conjunctivitis/keratitis, acne, otitis media/externa, pharyngitis and tonsillitis, epistaxis, and acute respiratory tract infection) with at least five cases in the population with AD. Medical conditions were considered chronic if included in the list of 114 EDCs defined by Salisbury et al. as those conditions that normally last six months or more, including past conditions that require ongoing disease or risk management, important conditions with a significant risk of recurrence, or past conditions that have continuing implications for patient management [21]. Multimorbidity was defined as the co-occurrence of two or more conditions from said list.

### 2.3. Statistical Analysis

We performed a descriptive analysis of the frequency and prevalence (%) of AD in children based on sex, age (i.e., 0–2-, 3–9-, 10–14-, and 15–17-year-olds), nationality, and residence area. We used logistic regression models to calculate the likelihood of presenting AD according to each of the categories of the aforementioned variables in the form of crude and age- and sex-adjusted odds ratios (ORs). Adjusted ORs were compared setting statistical significance at *p* < 0.05. We then analyzed the socio-demographic characteristics and comorbidity profile of children affected by AD. Results were calculated as means and/or frequencies with their corresponding standard deviations and/or 95% confidence intervals (CI).

For the analysis of AD comorbidity, we described the frequency and prevalence of chronic diseases and of the acute conditions of interest. For the identification of those comorbidities systematically associated with AD, we used logistic regression models to calculate the occurrence risk of each comorbidity (dependent variable) based on the presence or absence of AD (independent variable). We obtained crude and adjusted ORs for sex, age, deprivation index, and rurality. We compared adjusted ORs using the Bonferroni correction method for multiple comparisons to control familywise error rates (for a total of 82 comparisons of diseases with at least five cases), setting statistical significance at *p* < 0.00061. Statistical analyses were conducted using Stata software (Version 12.0, StataCorp LLC, College Station, TX, US).

## 3. Results

### 3.1. Prevalence and Socio-Demographics of Atopic Dermatitis in Children

A total of 33,591 children under 18 years of age belonging to the cohort showed an AD diagnosis during the study period, which resulted in an overall AD prevalence of 15.5% (Table 1). This prevalence was slightly higher in girls compared to boys (15.8% vs. 15.3%; OR 1.04, 95% CI 1.01–1.06), and in 3–9- (20.8%; OR 1.98, 95% CI 1.91–2.06) and 10–14-year-old children (14.0%; OR 1.26, 95% CI 1.21–1.31) compared to those aged 0–2 years (Table 2), although 15–17-year-old adolescents showed the lowest prevalence rate (7.8%; OR 0.64, 95% CI 0.61–0.67). Spanish children were more frequently affected by AD than those of different nationalities (ORs from 0.51 to 0.79). Regarding the area of residence, AD prevalence was slightly lower in rural areas (OR 0.95, 95% CI 0.93–0.97) and in the most deprived ones (OR 0.86, 95% CI 0.84–0.89). The mean age of children with AD was 7.72 years (SD 4.26), and the proportion of boys and girls was similar (Table 3).

### 3.2. Comorbidity of Atopic Dermatitis in Children

Over 40% of children with AD showed multimorbidity, with a mean disease burden of 1.62 (SD 0.88) chronic diseases (Table 3). The most frequent chronic comorbidities in children with AD of all ages and for both sexes were asthma (13.1%), psychosocial disorders (7.9%), visual impairment (7.8%), congenital anomalies of limbs (5.8%), and developmental disorders (3.2%), among others. All these comorbidities were more prevalent in the population with AD (Table 4). Upper respiratory tract infections (54.4%), otitis media (12.8%), conjunctivitis (10.1%), and pharyngitis (7.9%) were amongst the most prevalent acute conditions in this atopic cohort. The prevalence of both acute and chronic comorbidities varied based on sex and age group (Table S1, Supplementary Materials), with notable differences such as the earlier and more frequent occurrence of asthma, psycho-physiologic and somatoform disorders in boys than girls. 

Regardless of their prevalence and after adjusting by sex, age, rurality, and deprivation index, the conditions that AD was significantly associated to the most were the following: (OR (95% CI)) asthma (2.10 (2.02–2.17)), allergic rhinitis (2.00 (1.91–2.10)), and irritable bowel syndrome (1.90 (1.56–2.31)). A large and diverse number of comorbidities (i.e., cutaneous, neuropsychological, musculoskeletal) were also significantly more prevalent in the atopic population (Table 4). Chronic cutaneous disorders most associated to AD included psoriasis (1.62 (1.30–2.02)) and diseases of hair and hair follicles, including alopecia and seborrheic dermatitis (1.51 (1.38–1.64)). Childhood psychosocial disorders (1.53 (1.46–1.60)), developmental disorders (1.36 (1.27–1.45)), attention deficit disorder (1.37 (1.24–1.52)), and nonorganic sleep disorders (1.72 (1.55–1.90)) were the neuropsychological conditions most associated with AD. Impulse control (1.56 (1.13–2.15)), psycho-physiologic and somatoform disorders (1.47 (1.05–2.07)), and eating disorder (1.43 (1.12–1.83)) also presented high ORs, although not significant after applying the Bonferroni correction. Musculoskeletal comorbidities most frequent in AD children included congenital anomalies of limbs, hands, and feet (1.53 (1.45–1.61)), kyphoscoliosis (1.32 (1.22–1.42)), degenerative joint disease (1.42 (1.24–1.64)), and lower back pain (1.38 (1.15–1.65)). Metabolic diseases such as obesity (1.18 (1.07–1.29)) and disorders of lipid metabolism (1.46 (1.32–1.63)) were also significantly more prevalent in the atopic population, whereas cardiac ones such as congenital heart disease (1.25 (1.09–1.43)), cardiac valve disorders (1.57 (1.12–2.20)), and congestive heart failure (1.56 (1.00–2.43)), as well as hematologic disorders like lymphoma and anemia (1.40 (1.14–1.71)), presented high although not significant ORs.

## 4. Discussion

In this large-scale population-based study, we used real-world data to analyze AD prevalence in 216,291 pediatric users of the Spanish public health system. We exhaustively characterized the comorbidities of all 33,591 boys and girls with AD in the EpiChron Cohort, with a focus on the most prevalent chronic diseases and on those that were associated the most to AD regardless of their prevalence.

The prevalence of AD obtained in our population (15.5%) was similar to that found in other industrialized countries such as Sweden and Japan (16%) [7]. The soundest study to date in Spain on AD prevalence in children reported slightly higher rates in 6–7-year-olds (28%) than the ones shown by 3–9-year-olds in our population (22%) [8]. However, while the former study used surveys completed by children’s parents, we analyzed patients’ clinical histories to extract professionally diagnosed cases of AD, which could explain the variations in prevalence. Concurring with previous studies, and although prevalence differences between boys and girls were minimal in our population, girls were more affected by AD than boys [22,23].

Surprisingly, AD prevalence was similar in native (Spanish) and Asian or sub-Saharan African children, who come from areas of traditionally low prevalence [7]. Our results seem to support the theory that environmental factors related to industrialization could increase the risk of AD in people who migrate to industrialized countries [24]. However, we had no data on the immigration status of children or the age at which they migrated to Spain. This hypothesis also concurs with the well-known “hygiene theory” and with the slightly higher AD prevalence rates found in children from urban areas compared to rural environments [23,25]. Similarly, our findings, which reflected higher AD prevalence rates in children belonging to higher socio-economic levels, were consistent with the results reported in seven-year-old children in Great Britain, who also showed differences in this regard [26]. One might expect that more readily available access to healthcare could account for the observed increase in the prevalence of AD in children from urban and/or less deprived settings. However, the public health system in Spain provides equal conditions of access to care regardless of the area and, therefore, we believe that said increase in prevalence would be due to other factors.

In order to offer a global characterization of the comorbidity profile present in these children, we followed a two-pronged approach; we described both the most common comorbidities irrespective of their association to AD, as well as those with an increased risk of occurrence in children with a diagnosis of AD. The co-occurrence of these comorbidities could be a cause or a consequence of AD or share with it a common etiological mechanism. In line with previous studies, our investigation evidenced that children and adolescents with AD frequently suffer from multimorbidity (43%) and show an increased risk of developing a great number of diseases and symptoms of varying natures.

Prevalence rates of asthma, the most frequent AD comorbidity in our population, were lower (13%) than those reported in 3–5-year-olds in two Italian studies (24%) [27], and in 17-year-olds and under in the USA (25%) [28]. Furthermore, we found that atopy-related comorbidity prevalence rates increased with age; asthma and allergic reactions debuted at ages 3–9, followed by allergic rhinitis in the group of 10–14-year-olds in a sequence of increasing prevalence rates for different atopic conditions known as the “atopic march” [29]. 

In consonance with several studies highlighting the importance of mental health disorders [30,31,32,33] and suicidal ideation [34] in children with AD, psychosocial disorders were the second most prevalent chronic condition (7.9%) in our study population. Although the determining factors of this association are diverse, previous studies suggest that sleep disturbances play a key role in mental health disorder development in children with AD [31,35,36,37], particularly in conditions such as ADHD [38]. ADHD, in fact, was also more frequent in our study in children with AD compared to those without. These and other psychiatric disorders can be present even in preschool children with atopic eczema, although in our sample they become a relevant comorbidity in 10-year-olds and above [39].

Regarding visual impairment, the third most frequent comorbidity in our study (7.8%), Pols et al. [40] found an association between AD (OR (95% CI)) and blepharitis/styes/chalazion (1.53 (1.29–1.81)), infectious conjunctivitis (1.53 (1.20–1.81)), and allergic conjunctivitis (1.99 (1.59–2.49)) after analyzing a nationwide primary care database of children in the Netherlands. However, since the exact prevalence of each of the diagnoses included in the visual impairment EDC was unavailable, we were unable to contrast their results with our own. Our results for irritable bowel syndrome (1.90 (1.56–2.31)) were consistent with other recently published studies on children and adults with AD [41,42]. 

Musculoskeletal comorbidities, such as congenital anomalies of limbs, hands, and feet, kyphoscoliosis, degenerative joint disease, and lower back pain were more frequent in AD pediatric patients, who were shown to share common risk factors for injuries, including the aforementioned sleep disturbances, distractions due to chronic itch, the sedative effects of antihistamine drugs, and psychological comorbidities [43]. Additionally, low vitamin D blood levels in AD patients might also contribute to the skeletal burden [44]. Pols et al. found a higher prevalence of foot/toe-related symptoms (1.39 (1.20–1.60)) and acquired deformities of the limbs (1.39 (1.20–1.60)) in their study [40]; however, the association between AD and congenital limb anomalies or kyphoscoliosis was not established. 

Otological conditions, both acute and chronic, were also more prevalent in patients with AD. Otitis media was present in 13% of patients in our sample and was especially significant in 0–9-year-olds, showing higher rates than those found by Silverberg et al. in the United States of America (USA) (5%) [45]. A recent systematic review also found a higher risk of infectious otitis in patients with AD [45], whereas deafness and hearing loss, which had an adjusted OR of 1.52 (1.39–1.66) in our sample, had not been previously associated with AD. 

The prevalence of autoimmune diseases is reportedly higher in children with AD [46]. Alopecia areata frequently shows the strongest association [46,47], which would be consistent with the higher prevalence of hair and hair follicles diseases found in our sample. Psoriasis was also more prevalent in our population with AD, as previously described by Pols et al. [40]. The association of central obesity with AD was already reported, especially in women [16]. A recent study published by our group on 14-year-olds and below concluded that the prevalence of overweight, obesity, and dyslipidemia was greater in those with AD [48], which concurs with our present findings.

We have to highlight the existence of some comorbidities of varying nature that, although not significant after applying the Bonferroni correction for multiple comparisons, deserve special attention since they could be potentially associated with AD, as they presented some of the highest ORs of our study. This is the case of chronic cystic disease of the breast (2.61 (1.13–6.06)) that, despite its very low prevalence (0.02%), was the condition AD was associated to the most, and which had not been previously reported in other studies that we know of. Congenital heart diseases and cardiac valve disorders also presented high ORs and had not been described in the existing literature either. As certain authors already suggested, AD could potentially constitute a risk factor for the development of cardiovascular diseases during adulthood [15,49]. Inflammatory bowel disease, although infrequent, also showed one of the highest adjusted ORs (1.86 (1.01–3.45)). Previous studies support the association of both diseases in children [46,50] and, in fact, inflammatory bowel disease shares 39 genetic risk loci with AD, also suggesting a genetic cause [51].

Our results could help better understand the comorbidity of AD and improve its overall management and treatment, providing patients with holistic and person-centered care. Nonetheless, statistical associations do not necessarily translate into the existence of causal relationships, and these findings should be interpreted with caution. Although many common underlying factors are well documented in the existing literature, some of the associations found in our study were not previously considered and could set the basis for future studies aimed at identifying common pathophysiological pathways connecting AD to other conditions of varying nature. 

The main strength of our work lies in its large-scale, population-based characteristics, as it included almost every child with an AD diagnosis belonging to the reference population area. Moreover, AD comorbidity was exhaustively analyzed through the study of every chronic disease diagnosis in both primary and hospital care, not only the most prevalent or relevant ones. The use of EHRs guaranteed the reliability of data, which underwent continuous quality control revisions to ensure its accuracy. The main limitation of our study was its cross-sectional nature, which made determining the chronological order of appearance of diseases impossible, thus hindering the identification of cause–effect relationships between AD and its comorbidities. Furthermore, EHRs were not specifically designed for the purposes of this research, thus leading to potential over- or under-reporting of certain diseases. Moreover, some variables of interest for the study, such as lifestyle habits (e.g., lack of exercise, unhealthy eating), environmental information (e.g., air pollution, weather conditions), or specific data on immigration status of children were not available in the cohort.

## 5. Conclusions

The present study evidences that AD is prevalent in children and that it is systematically associated with some relevant non-dermatological conditions of neuropsychiatric, visual, cardio-metabolic, and autoimmune nature, among others. Our results highlight the importance of monitoring the array of comorbidities surrounding AD during childhood, which could help improve their clinical management and plan interventions aimed at preventing their development. Future longitudinal studies are encouraged to shed light on the underlying pathophysiological mechanisms involved in these associations.

## Figures and Tables

**Table 1:** Frequency and prevalence (%) of atopic dermatitis (AD) in children of the EpiChron Cohort aged 0–17 years in 2015 according to sex, age, nationality, area of residence and deprivation index. EU—European Union.

	Boys (*n* = 110,934)	Girls (*n* = 105,357)	Total (*n* = 216,291)
AD Prevalence	*n* (%)	*n* (%)	*n* (%)
Sex	16,968 (15.3)	16,623 (15.8)	33,591 (15.5)
Age (years)			
0–2	2110 (12.5)	1704 (10.7)	3814 (11.7)
3–9	9320 (20.5)	9230 (21.1)	18,550 (20.8)
10–14	4225 (13.7)	4358 (14.8)	8583 (14.2)
15–17	1313 (7.42)	1331 (8.19)	2644 (7.79)
Nationality			
Spain	15,184 (16.1)	14,863 (16.6)	30,047 (16.3)
Eastern Europe	685 (10.0)	714 (10.8)	1399 (10.4)
Asia	150 (13.0)	145 (13.6)	295 (13.3)
North Africa	459 (11.6)	423 (11.4)	882 (11.5)
Sub-Saharan Africa	201 (13.3)	197 (14.9)	398 (14.1)
Latin America	232 (10.2)	223 (9.98)	455 (10.1)
EU and North America	52 (8.54)	54 (8.72)	106 (8.63)
Area of residence ^1^			
Urban	10,297 (15.5)	10,321 (16.2)	20,496 (15.7)
Rural	6671 (15.0)	6302 (15.2)	13,095 (15.2)
Deprivation index ^2^			
Q_1_	5259 (16.4)	5026 (16.4)	10,285 (16.4)
Q_2_	4085 (15.3)	4112 (16.0)	8197 (15.7)
Q_3_	3356 (15.4)	3316 (16.2)	6672 (15.8)
Q_4_	4268 (14.0)	4169 (14.6)	8437 (14.3)

^1^ Based on the type of administrative health area (urban, if 80% or more of the population of the area is concentrated in one of the municipalities, or rural for the rest); ^2^ calculated at aggregated level per administrative health area according to 26 socio-economic indicators and divided into quartiles from less (Q1) to more (Q4) deprived.

**Table 2:** Likelihood of presenting atopic dermatitis based on sex, age, nationality, area of residence (urban/rural), and deprivation index of the area, calculated using logistic regression models.

Variable	Crude OR ^1^	*p*-Value ^2^	Adjusted OR ^3^	*p*-Value
Sex				
Boys	Reference			
Girls	1.04 (1.01–1.06)	0.002		
Age (years)				
0–2	Reference			
3–9	1.98 (1.91–2.06)	<0.001		
10–14	1.26 (1.21–1.31)	<0.001		
15–17	0.64 (0.61–0.67)	<0.001		
Nationality				
Spain	Reference		Reference	
Sub-Saharan Africa	0.84 (0.76–0.94)	0.001	0.79 (0.71–0.88)	<0.001
Asia	0.78 (0.69–0.89)	<0.001	0.76 (0.67–0.86)	<0.001
Eastern Europe	0.59 (0.56–0.63)	<0.001	0.59 (0.56–0.62)	<0.001
Latin America	0.57 (0.52–0.63)	<0.001	0.61 (0.55–0.67)	<0.001
North Africa	0.67 (0.62–0.72)	<0.001	0.63 (0.58–0.67)	<0.001
EU and North America	0.48 (0.40–0.59)	<0.001	0.51 (0.41–0.62)	<0.001
Area of residence				
Urban	Reference		Reference	
Rural	0.95 (0.93–0.97)	<0.001	0.95 (0.93–0.97)	<0.001
Deprivation index ^4^				
Q_1_	Reference		Reference	
Q_2_	0.95 (0.92–0.98)	0.001	0.97 (0.94–1.00)	0.081
Q_3_	0.96 (0.92–0.99)	0.008	0.97 (0.94–1.00)	0.099
Q_4_	0.85 (0.82–0.88)	<0.001	0.86 (0.84–0.89)	<0.001

^1^ Odds ratio; ^2^ *p*-values less than 0.05 were considered significant and are highlighted in bold; ^3^ adjusted odds ratios for age and sex; ^4^ calculated at aggregated level per administrative health area according to 26 socio-economic indicators and divided into quartiles from less (Q1) to more (Q4) deprived.

**Table 3:** Socio-demographic and clinical characteristics of children of the EpiChron Cohort aged 0–17 years with atopic dermatitis in 2015.

Characteristics	Boys	Girls	Total
*n* (%)	16,968 (50.5)	16,623 (49.5)	33,591 (100)
Mean age, years (SD ^1^)	7.61 (4.29)	7.83 (4.24)	7.72 (4.26)
Age group, years (*n*, %)			
0–2	2110 (12.4)	1704 (10.2)	3814 (11.3)
3–9	9320 (54.9)	9230 (55.5)	18,550 (55.2)
10–14	4225 (24.9)	4358 (26.2)	8583 (25.6)
15–17	1313 (7.74)	1331 (8.01)	2644 (7.87)
Nationality (*n*, %)			
Spain	15,184 (89.5)	14,863 (89.4)	30,047 (89.5)
Eastern Europe	685 (4.04)	714 (4.30)	1399 (4.17)
Asia	150 (0.88)	145 (0.87)	295 (0.88)
North Africa	459 (2.71)	423 (2.55)	882 (2.63)
Sub-Saharan Africa	201 (1.18)	197 (1.19)	398 (1.19)
Latin America	232 (1.37)	223 (1.34)	455 (1.35)
EU and North America	52 (0.31)	54 (0.32)	106 (0.32)
Area of residence			
Urban ^2^ (*n*, %)	10,297 (60.7)	10,321 (62.1)	20,618 (61.4)
Deprivation index ^3^ (*n*, %)			
Q_1_	5259 (31.0)	5026 (30.2)	10,285 (30.6)
Q_2_	4085 (24.1)	4112 (24.7)	8197 (24.4)
Q_3_	3356 (19.8)	3316 (19.9)	6672 (19.9)
Q_4_	4268 (25.1)	4169 (25.1)	8437 (25.1)
Number of chronic diseases (mean, SD)	1.67 (0.91)	1.58 (0.85)	1.62 (0.88)
Multimorbidity ^4^, yes (*n*, %)	7773 (45.8)	6794 (40.9)	(43.4)

^1^ Standard deviation; ^2^ versus rural; ^3^ calculated at aggregated level per administrative health area according to 26 socio-economic indicators and divided into quartiles from less (Q1) to more (Q4) deprived; ^4^ defined as the presence of two or more chronic diseases from a list of 114 conditions.

**Table 4:** Prevalence of chronic comorbidities and of specific acute conditions (in italics) in children aged 0–17 years with atopic dermatitis (AD) in the EpiChron Cohort in 2015 (*n* = 33,591). Logistic regression models were used to calculate odds ratios (OR) of prevalence for each comorbidity (dependent variable) according to the presence or not of AD (independent variable). HIV—human immunodeficiency virus; AIDS—acquired immune deficiency syndrome.

EDC ^1^	Comorbidity	Prevalence ^2^	Crude OR ^3^	Adjusted OR ^5^	*p*-Value ^6^
		(*n*, %)	(95% CI ^4^)	(95% CI)	
*EAR11*	*Acute upper respiratory tract infection*	18,256 (54.4)	1.52 (1.48–1.55)	1.42 (1.39–1.46)	0.0000
ALL04/05	Asthma	4384 (13.05)	1.94 (1.87–2.01)	2.10 (2.02–2.17)	0.0000
*EAR01*	Otitis media	4287 (12.8)	1.52 (1.47–1.58)	1.45 (1.40–1.51)	0.0000
*EYE07*	Conjunctivitis, keratitis	3388 (10.1)	1.45 (1.39–1.51)	1.39 (1.33–1.44)	0.0000
*EAR09*	Pharyngitis and tonsillitis	2662 (7.92)	1.67 (1.60–1.75)	1.74 (1.67–1.83)	0.0000
PSY14	Psychosocial disorders of childhood	2640 (7.86)	1.34 (1.28–1.40)	1.53 (1.46–1.60)	0.0000
EYE02	Visual impairment	2621 (7.80)	1.24 (1.19–1.30)	1.48 (1.42–1.55)	0.0000
*ALL03*	Allergic rhinitis	2386 (7.10)	1.50 (1.43–1.57)	2.00 (1.91–2.10)	0.0000
MUS11	Congenital anomalies of limbs	1952 (5.81)	1.53 (1.46–1.61)	1.53 (1.45–1.61)	0.0000
*EAR06*	Otitis externa	1197 (3.56)	1.49 (1.39–1.59)	1.47 (1.37–1.56)	0.0000
NUR19	Developmental disorder	1080 (3.22)	1.36 (1.27–1.45)	1.36 (1.27–1.45)	0.0000
*SKN04*	Acne	940 (2.80)	0.74 (0.70–0.80)	1.18 (1.10–1.27)	0.0000
MUS06	Kyphoscoliosis	816 (2.43)	0.88 (0.82–0.95)	1.32 (1.22–1.42)	0.0000
SKN13	Disease of hair and hair follicles	684 (2.04)	1.31 (1.21–1.43)	1.51 (1.38–1.64)	0.0000
END05	Other endocrine disorders	671 (2.00)	1.04 (0.96–1.13)	1.21 (1.12–1.32)	0.0000
EAR08	Deafness, hearing loss	652 (1.94)	1.41 (1.29–1.54)	1.52 (1.39–1.66)	0.0000
PSY19	Sleep disorders, nonorganic origin	519 (1.55)	1.76 (1.59–1.94)	1.72 (1.55–1.90)	0.0000
PSY05	Attention deficit disorder	484 (1.44)	1.03 (0.94–1.14)	1.37 (1.24–1.52)	0.0000
CAR11	Disorders of lipid metabolism	422 (1.26)	1.21 (1.09–1.34)	1.46 (1.32–1.63)	0.0000
EAR10	Epistaxis	331 (0.99)	1.56 (1.38–1.76)	1.57 (1.39–1.78)	0.0000
MUS03	Degenerative joint disease	238 (0.71)	1.02 (0.89–1.17)	1.42 (1.24–1.64)	0.0000
GAS09	Irritable bowel syndrome	132 (0.39)	1.83 (1.51–2.24)	1.90 (1.56–2.31)	0.0000
SKN12	Psoriasis	102 (0.30)	1.24 (1.00–1.54)	1.62 (1.30–2.02)	0.0000
RES04	COPD ^7^	66 (0.20)	1.72 (1.30–2.27)	1.76 (1.33–2.32)	0.0001
NUT03	Obesity	566 (1.68)	0.91 (0.84–1.00)	1.18 (1.07–1.29)	0.0005
MUS14	Lower back pain	141 (0.42)	0.91 (0.76–1.08)	1.38 (1.15–1.65)	0.0006
HEM08	Hematologic disorders, other	118 (0.35)	1.39 (1.14–1.71)	1.40 (1.14–1.71)	0.0012
CAR04	Congenital heart disease	250 (0.74)	1.31 (1.14–1.50)	1.25 (1.09–1.43)	0.0017
END04	Hypothyroidism	167 (0.50)	1.02 (0.86–1.20)	1.28 (1.08–1.52)	0.0036
PSY15	Eating disorder	79 (0.24)	1.13 (0.88–1.43)	1.43 (1.12–1.83)	0.0041
PSY16	Impulse control	47 (0.14)	1.22 (0.89–1.68)	1.56 (1.13–2.15)	0.0066
CAR06	Cardiac valve disorders	44 (0.13)	1.48 (1.06–2.06)	1.57 (1.12–2.20)	0.0083
RES11	Respiratory disorders, other	118 (0.35)	1.37 (1.12–1.68)	1.31 (1.07–1.61)	0.0087
GSU06	Chronic cystic disease of the breast	7 (0.02)	1.36 (0.59–3.11)	2.61 (1.13–6.06)	0.0253
PSY17	Psycho-physiological and somatoform disorders	42 (0.13)	1.28 (0.92–1.80)	1.47 (1.05–2.07)	0.0255
GAS02	Inflammatory bowel disease	13 (0.04)	1.31 (0.71–2.40)	1.86 (1.01–3.45)	0.0473
CAR05	Congestive heart failure	25 (0.07)	1.40 (0.90–2.18)	1.56 (1.00–2.43)	0.0496
CAR09	Cardiac arrhythmia	69 (0.21)	1.20 (0.92–1.55)	1.29 (0.99–1.67)	0.0599
GUR10	Prostatitis	14 (0.04)	1.77 (0.97–3.24)	1.77 (0.97–3.25)	0.0635
GTC01	Chromosomal anomalies	79 (0.24)	1.23 (0.96–1.57)	1.25 (0.98–1.60)	0.0729
MAL03	High impact malignant neoplasms	93 (0.28)	1.03 (0.82–1.28)	1.22 (0.98–1.53)	0.0805
EYE13	Diabetic retinopathy	16 (0.05)	1.67 (0.96–2.93)	1.64 (0.93–2.87)	0.0849
END06–09	Diabetes	81 (0.24)	1.09 (0.85–1.38)	1.23 (0.97–1.56)	0.0923
GSU08	Varicose veins of lower extremities	17 (0.05)	1.13 (0.67–1.90)	1.55 (0.91–2.63)	0.1053
NUR21	Neurologic disorders, other	200 (0.60)	1.12 (0.96–1.31)	1.13 (0.97–1.32)	0.1114
GUR03	Hypospadias, penile anomalies	106 (0.32)	1.23 (1.00–1.52)	1.19 (0.96–1.47)	0.1116
MAL12	Malignant neoplasms, colorectal	1 (0.00)	0.21 (0.03–1.54)	0.21 (0.03–1.55)	0.1258
END02	Osteoporosis	5 (0.01)	1.60 (0.59–4.34)	2.15 (0.78–5.92)	0.1387
HEM01	Hemolytic anemia	86 (0.26)	1.09 (0.87–1.38)	1.19 (0.94–1.50)	0.1515
REN04	Nephritis, nephrosis	36 (0.11)	1.18 (0.82–1.69)	1.29 (0.90–1.85)	0.1698
CAR14/15	Hypertension	37 (0.11)	1.01 (0.71–1.43)	1.28 (0.90–1.82)	0.1770
MAL01	Malignant neoplasms of skin	8 (0.02)	1.40 (0.65–3.05)	1.69 (0.77–3.70)	0.1927
NUR08	Multiple sclerosis	5 (0.01)	1.81 (0.66–4.99)	1.95 (0.70–5.40)	0.1995
GAS08	Gastroesophageal reflux	7 (0.02)	0.62 (0.29–1.36)	0.60 (0.28–1.32)	0.2046
RHU03	Arthropathy	7 (0.02)	1.81 (0.77–4.27)	1.72 (0.73–4.04)	0.2157
INF04	HIV infection, AIDS	19 (0.06)	1.40 (0.84–2.31)	1.37 (0.83–2.27)	0.2190
HEM03	Thrombophlebitis	7 (0.02)	0.56 (0.26–1.22)	0.62 (0.28–1.36)	0.2332
RES06	Sleep apnea	12 (0.04)	1.48 (0.78–2.81)	1.43 (0.75–2.71)	0.2731
PSY08	Personality disorders	41 (0.12)	0.80 (0.58–1.12)	1.16 (0.84–1.62)	0.3687
GSU11	Peripheral vascular disease	7 (0.02)	1.12 (0.50–2.53)	1.44 (0.63–3.28)	0.3872
ALL06	Disorders of the immune system	34 (0.10)	1.12 (0.77–1.62)	1.15 (0.79–1.67)	0.4602
CAR03	Ischemic heart disease (excluding AMI ^8^)	17 (0.05)	0.86 (0.52–1.44)	0.83 (0.50–1.39)	0.4847
REC03	Chronic ulcer of the skin	10 (0.03)	0.73 (0.38–1.42)	0.79 (0.41–1.54)	0.4899
NUR07	Seizure disorder	222 (0.66)	1.01 (0.87–1.16)	1.05 (0.91–1.21)	0.5276
GTC02	Inherited metabolic disorders	5 (0.01)	0.80 (0.31–2.05)	0.76 (0.30–1.95)	0.5685
HEM02	Iron deficiency anemia	253 (0.75)	0.96 (0.84–1.10)	1.04 (0.91–1.19)	0.5794
REN05	Renal disorders, other	26 (0.08)	1.02 (0.67–1.56)	1.13 (0.74–1.72)	0.5819
PSY07	Schizophrenia, affective psychosis	51 (0.15)	0.99 (0.74–1.34)	1.09 (0.81–1.47)	0.5829
PSY02	Substance use	35 (0.10)	0.71 (0.50–1.01)	1.09 (0.76–1.56)	0.6293
HEM07	Hemophilia, coagulation disorder	6 (0.02)	1.21 (0.50–2.93)	1.20 (0.49–2.91)	0.6909
NUR03	Peripheral neuropathy, neuritis	43 (0.13)	0.90 (0.65–1.25)	0.94 (0.68–1.30)	0.7011
EYE06	Cataract, aphakia	9 (0.03)	0.83 (0.41–1.67)	0.87 (0.43–1.77)	0.7051
NUR16	Spinal cord injury/disorders	11 (0.03)	0.92 (0.49–1.74)	0.89 (0.47–1.68)	0.7091
MAL16	Acute leukemia	8 (0.02)	0.78 (0.37–1.63)	0.88 (0.42–1.86)	0.7403
PSY09	Depression	44 (0.13)	0.67 (0.49–0.92)	1.05 (0.76–1.44)	0.7751
CAR16	Cardiovascular disorders, other	79 (0.24)	0.96 (0.76–1.22)	1.04 (0.81–1.32)	0.7795
GUR09	Renal calculi	14 (0.04)	1.02 (0.57–1.80)	1.08 (0.61–1.93)	0.7817
ADM02	Surgical aftercare	25 (0.07)	0.99 (0.64–1.51)	1.06 (0.69–1.63)	0.7922
NUR05	Cerebrovascular disease	22 (0.07)	1.04 (0.66–1.64)	1.04 (0.66–1.64)	0.8678
GUR01	Vesicoureteral reflux	6 (0.02)	0.99 (0.41–2.36)	1.07 (0.45–2.58)	0.8768
INF01	Tuberculosis infection	79 (0.24)	0.88 (0.69–1.11)	1.01 (0.80–1.29)	0.9155
PSY01	Anxiety, neuroses	72 (0.21)	0.69 (0.54–0.89)	1.00 (0.78–1.28)	0.9718

^1^ Expanded diagnostic clusters; ^2^ comorbidities appear in descending order of the *p*-value of the comparison; only diseases with a frequency of five or more cases are represented; ^3^ confidence interval; ^4^ unadjusted odds ratios; ^5^ odds ratios adjusted by sex, age, deprivation index and area (rural/urban); ^6^ *p*-values for the adjusted OR; *p*-values < 0.00061 are highlighted in bold as significant according to Bonferroni correction for multiple comparisons; ^7^ chronic obstructive pulmonary disease; ^8^ acute myocardial infarction.

## Supplementary Materials

The following are available online at https://www.mdpi.com/2077-0383/9/6/1632/s1: Table S1. Prevalence of the 10 most frequent diseases (both acute or chronic) in children with atopic dermatitis, according to age and sex.
