# Supplementary material for: Prevalence and Comorbidity of Atopic Dermatitis in Children: A Large-Scale Population Study Based on Real-World Data
Source: J Clin Med. 2020 May 28;9(6):1632. doi: 10.3390/jcm9061632 (PMC7356227; doi:10.3390/jcm9061632)
Supplement: Supplementary file 1 [file jcm-09-01632-s001.pdf]

Table 1. Prevalence of the ten most frequent diseases (both acute or chronic) in children with atopic dermatitis, according to age and sex.

**Sex**	**Boys**		**Girls**		**Total**	
**Age interval**	**Comorbidity**	**n (%)**	**Comorbidity**	**n (%)**	**Comorbidity**	**n (%)**
**0 - 2 years**	Acute upper respiratory tract infection	1753 (83.08)	Acute upper respiratory tract infection	1390 (81.57)	Acute upper respiratory tract infection	3143 (82.41)
	Fever	898 (42.56)	Fever	703 (41.26)	Fever	1601 (41.98)
	Acute lower respiratory tract infection	782 (37.06)	Acute lower respiratory tract infection	539 (31.63)	Acute lower respiratory tract infection	1321 (34.64)
	Gastroenteritis	582 (27.58)	Gastroenteritis	443 (26.00)	Gastroenteritis	1025 (26.87)
	Otitis media	562 (26.64)	Cough	429 (25.18)	Cough	980 (25.69)
	Conjunctivitis, keratitis	557 (26.40)	Otitis media	402 (23.59)	Otitis media	964 (25.28)
	Cough	551 (26.11)	Conjunctivitis, keratitis	386 (22.65)	Conjunctivitis, keratitis	943 (24.72)
	Gastrointestinal signs and symptoms	387 (18.34)	Gastrointestinal signs and symptoms	276 (16.20)	Gastrointestinal signs and symptoms	663 (17.38)
	Exanthems	353 (16.73)	Nonspecific signs and symptoms	267 (15.67)	Exanthems	620 (16.26)
	Nonspecific signs and symptoms	339 (16.07)	Exanthems	267 (15.67)	Nonspecific signs and symptoms	606 (15.89)
**3 - 9 years**	Acute upper respiratory tract infection	5214 (55.94)	Acute upper respiratory tract infection	5330 (57.75)	Acute upper respiratory tract infection	10544 (56.84)
	Fever	1933 (20.74)	Fever	1859 (20.14)	Fever	3792 (20.44)
	Cough	1753 (18.81)	Disorders of mouth	1620 (17.55)	Cough	3330 (17.95)
	Disorders of mouth	1559 (16.73)	Cough	1577 (17.09)	Disorders of mouth	3179 (17.14)
	Asthma, w/o status asthmaticus	1395 (14.97)	Otitis media	1413 (15.31)	Otitis media	2773 (14.95)
	Asthma	1395 (14.97)	Acute lower respiratory tract infection	1105 (11.97)	Acute lower respiratory tract infection	2476 (13.35)

	Acute lower respiratory tract infection	1371 (14.71)	Allergic reactions	1102 (11.94)	Allergic reactions	2356 (12.70)
	Otitis media	1360 (14.59)	Gastroenteritis	980 (10.62)	Asthma, w/o status asthmaticus	2336 (12.59)
	Allergic reactions	1254 (13.45)	Viral syndromes	975 (10.56)	Asthma	2336 (12.59)
	Gastroenteritis	1021 (10.95)	Asthma, w/o status asthmaticus	941 (10.20)	Gastroenteritis	2001 (10.79)
**10 - 14 years**	Acute upper respiratory tract infection	1696 (40.14)	Acute upper respiratory tract infection	1821 (41.79)	Acute upper respiratory tract infection	3517 (40.98)
	Asthma, w/o status asthmaticus	859 (20.33)	Musculoskeletal signs and symptoms	756 (17.35)	Musculoskeletal signs and symptoms	1467 (17.09)
	Asthma	859 (20.33)	Allergic reactions	627 (14.39)	Asthma, w/o status asthmaticus	1464 (17.06)
	Allergic reactions	712 (16.85)	Blindness	610 (14.00)	Asthma	1464 (17.06)
	Musculoskeletal signs and symptoms	711 (16.83)	Asthma, w/o status asthmaticus	605 (13.88)	Allergic reactions	1339 (15.60)
	Psycho-physiologic and somatoform disorders	688 (16.28)	Asthma	605 (13.88)	Allergic rhinitis	1146 (13.35)
	Allergic rhinitis	605 (14.32)	Allergic rhinitis	541 (12.41)	Blindness	1113 (12.97)
	Blindness	503 (11.91)	Nonspecific signs and symptoms	516 (11.84)	Psycho-physiologic and somatoform disorders	1085 (12.64)
	Nonspecific signs and symptoms	483 (11.43)	Refractive errors	465 (10.67)	Nonspecific signs and symptoms	999 (11.64)
	Chronic pharyngitis and tonsillitis	447 (10.58)	Abdominal pain	445 (10.21)	Refractive errors	841 (9.80)
**15 - 17 years**	Acute upper respiratory tract infection	492 (37.47)	Acute upper respiratory tract infection	560 (42.07)	Acute upper respiratory tract infection	1052 (39.79)

	Allergic rhinitis	233 (17.75)	Acne	223 (16.75)	Asthma, w/o status asthmaticus	432 (16.34)
	Asthma, w/o status asthmaticus	230 (17.52)	Musculoskeletal signs and symptoms	208 (15.63)	Asthma	432 (16.34)
	Asthma	230 (17.52)	Asthma, w/o status asthmaticus	202 (15.18)	Allergic rhinitis	426 (16.11)
	Allergic reactions	190 (14.47)	Asthma	202 (15.18)	Acne	398 (15.05)
	Musculoskeletal signs and symptoms	184 (14.01)	Allergic rhinitis	193 (14.50)	Musculoskeletal signs and symptoms	392 (14.83)
	Refractive errors	175 (13.33)	Blindness	193 (14.50)	Allergic reactions	380 (14.37)
	Acne	175 (13.33)	Allergic reactions	190 (14.27)	Blindness	343 (12.97)
	Psycho-physiologic and somatoform disorders	173 (13.18)	Refractive errors	165 (12.40)	Refractive errors	340 (12.86)
	Blindness	150 (11.42)	Kyphoscoliosis	142 (10.67)	Psycho-physiologic and somatoform disorders	266 (10.06)
**Total**	Acute upper respiratory tract infection	9155 (53.95)	Acute upper respiratory tract infection	9101 (54.75)	Acute upper respiratory tract infection	18256 (54.35)
	Fever	3219 (18.97)	Fever	2929 (17.62)	Fever	6148 (18.30)
	Cough	2722 (16.04)	Cough	2348 (14.13)	Cough	5070 (15.09)
	Asthma, w/o status asthmaticus	2589 (15.26)	Disorders of mouth	2164 (13.02)	Allergic reactions	4417 (13.15)
	Asthma	2589 (15.26)	Otitis media	2115 (12.72)	Asthma, w/o status asthmaticus	4384 (13.05)
	Acute lower respiratory tract infection	2436 (14.36)	Allergic reactions	2049 (12.33)	Asthma	4384 (13.05)
	Allergic reactions	2368 (13.96)	Musculoskeletal signs and symptoms	1992 (11.98)	Acute lower respiratory tract infection	4290 (12.77)

	Otitis media	2172 (12.80)	Acute lower respiratory tract infection	1854 (11.15)	Otitis media	4287 (12.76)
	Disorders of mouth	2089 (12.31)	Nonspecific signs and symptoms	1822 (10.96)	Disorders of mouth	4253 (12.66)
	Gastroenteritis	1936 (11.41)	Asthma, w/o status asthmaticus	1795 (10.80)	Musculoskeletal signs and symptoms	3872 (11.53)
